# Assessing vitamin D related genetic variants, status, and influence factors in pregnant women in Eastern and Central China

**DOI:** 10.1002/fsn3.1674

**Published:** 2020-06-18

**Authors:** Xiaofei Qiu, Xinhao Chen, Shangming Zuo, Yuan Ji, Zheng Wen, Linna Wei, Shouxin Wu, Le Diao, Bo Li, Jiangman Zhao, Tianrui Chen

**Affiliations:** ^1^ Department of Gynaecology and Obstetrics Central People's Hospital of Tengzhou Tengzhou China; ^2^ Endocrine Department of Nephropathy Central Hospital of Dengzhou Dengzhou China; ^3^ Department of Pediatrics Central Hospital of Dengzhou Dengzhou China; ^4^ Neonatal Intensive Care Center Central Hospital of Dengzhou Dengzhou China; ^5^ Zhangjiang Center for Translational Medicine Biotecan Medical Diagnostics Co., Ltd. Shanghai China; ^6^ Department of Orthopedic Oncology Changzheng Hospital Second Military Medical University Shanghai China

**Keywords:** 25(OH)D, liquid chromatography‐tandem mass spectrometry, pregnant women, single nucleotide polymorphisms, vitamin D deficiency

## Abstract

Vitamin D deficiency has recently become a global public health problem. However, it is still unclear if gene polymorphisms in the vitamin D pathway influence vitamin D levels among pregnant women in Eastern and Central China. The objective of this study was to assess factors influencing vitamin D levels in pregnant women. A total of 326 participants in Shandong and Henan provinces in China were enrolled from August 2017 to April 2019. Serum 25(OH)D levels and single nucleotide polymorphisms (SNPs) in the vitamin D pathway were measured using the blood samples collected in the first trimester, second trimester, and third trimester. Data on demographics, lifestyle, and health behavior were collected using a questionnaire. Statistical analyses were performed using the R software. The prevalence of 25(OH)D deficiency was significantly more severe in pregnant women. The average 25(OH)D value of all enrolled pregnant women was 14.57 ± 7.21 ng/ml (deficiency). Only 15 (4.60%) participants had a 25(OH)D concentration ≥30 ng/ml (sufficient). The prevalence of four ranks of vitamin D levels from severe 25(OH)D deficiency to 25(OH)D sufficiency (<10, 10–20, 20–30, and ≥30 ng/ml) was 29.14%, 52.45%, 13.80%, and 4.60%, respectively. Variants of GC (rs1155563) and CYP24A1 (rs6013897) were significantly associated with both 25(OH)D concentrations and vitamin D deficiency among pregnant women, respectively. Our findings suggest that pregnant women in Eastern and Central China are at high risk of vitamin D deficiency. Genetic mutants in the vitamin D pathway (GC and CYP24A1) were significantly associated with 25(OH)D levels in pregnant women in Eastern and Central China.

## Graphical abstract

Results of this study showed that pregnant women were at high risk of vitamin D deficiency in Eastern and Central China. Serum 25(OH)D level was influenced by BMI, physical activity time, vitamin D intake, and gene polymorphisms in vitamin D pathway (rs1155563 in GC and rs6013897 in CYP24A1) among pregnant women. Vitamin D supplementation could modify this effect of gene polymorphisms.

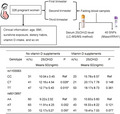

## 1 INTRODUCTION

Vitamin D plays an important role in regulating calcium and phosphorus metabolism and maintaining normal bone mineral homeostasis in the human body (Holick, 2007; White, 2008). The risk of osteomalacia and fracture is proportional to vitamin D deficiency (Bischoff‐Ferrari et al., 2009). Recent research has found that vitamin D deficiency is also related to immune dysfunction, tumors, cardiovascular diseases (Adams & Hewison, 2010), and blood glucose metabolic disorders (Leung, 2016). Vitamin D deficiency can cause systemic diseases and warrants attention as a global health problem. Nowadays, compared with men, women have fewer outdoor activities and often use sunscreen; thus, pregnant women's demand for vitamin D has increased significantly (Daly et al., 2012; Zhen, Liu, Guan, Zhao, & Tang, 2015), and vitamin D deficiency is more common in pregnant women. Vitamin D deficiency or insufficiency may be related to pregnancy complications and adverse pregnancy outcomes, including preeclampsia, gestational diabetes mellitus, and preterm birth (Bodnar et al., 2007, 2013; Brannon, 2012). As has been well researched, 25‐hydroxyvitamin D (25(OH)D) is currently regarded as the only representative biomarker of serum vitamin D and is supplied by cutaneous synthesis and dietary intake (Zerwekh, 2008).

Vitamin D is hydroxylated by cytochrome P450 vitamin D hydroxylases in the liver (CYP2R1, CYP24A1, and CYP3A43). The CYP27B1 (cytochrome P450 monooxygenase 25(OH)D 1αhydroxylase) that metabolizes 25(OH)D_3_ to 1,25(OH)_2_D_3_ is present in the kidney. Calcitriol conversed in the kidney binds to the vitamin D binding protein (DBP), which is encoded by the GC vitamin D binding protein (GC) gene. 24‐hydroxylase (CYP24A1) is capable of transforming 25(OH)D and 1,25(OH)_2_D to their inactive forms to protect target organs or tissues from excessive vitamin D signaling (Carter, 2011). Thus, the genes involved in vitamin D synthesis and metabolism pathways would influence the circulating level of vitamin D. Two genome‐wide association studies have found strong associations between polymorphisms of GC and CYP2R1 genes with vitamin D levels (Ahn et al., 2010; Wang, Zhang, et al., 2010). Variants near the genes involved in cholesterol synthesis, hydroxylation, and vitamin D transport affect the vitamin D status. Genetic variations at these loci could identify individuals with a high risk of vitamin D insufficiency (Wang, Zhang, et al., 2010). Barry et al. (2014) revealed that the increase in serum (25(OH)D) attributable to vitamin D_3_ supplementation may vary according to common genetic differences in vitamin D 25‐hydroxylase (CYP2R1), 24‐hydroxylase (CYP24A1), and the vitamin D receptor (VDR) genes. Other previous studies have also reported significant associations of variants in VDR, CYP27B1, CYP24A1, DHCR7/NADSYN1, CYP27A1, and CYP3A4 (Barry et al., 2014; Gupta, Patrick, & Bell, 2007; Lu, Sheng, et al., 2012), and LRP2 with vitamin D concentrations (Nykjaer et al., 1999).

Recent studies have found that pregnant women in South China are at high risk of vitamin D insufficiency (Wang, Yang, et al., 2010; Xiang et al., 2013; Xiao et al., 2015). However, there are very few studies on the association between genetic polymorphisms and vitamin D concentrations among pregnant women in China. Thus, we conducted a study among pregnant women in Eastern and Central China to evaluate the relationships between SNPs of vitamin D pathway genes and serum vitamin D levels in the first trimester, second trimester, and third trimester. Some studies have evaluated vitamin D status in healthy Chinese populations. For example, Hjelmesaeth, Roislien, Hofso, and Bollerslev's (2010) revealed the relationship between vitamin D and metabolic syndrome in middle‐aged and elderly Chinese individuals, while Foo et al. (2009) evaluated vitamin D levels in girls aged 12–14 years in Beijing. Another study described the vitamin D status in Chinese subjects by collecting data from five cities, namely Dalian, Beijing, Hangzhou, Guangzhou, and Urumqi (Yu et al., 2015). However, the status of vitamin D is mediated by different factors, including gender, age, region, ethnicity, clothing habits, and supplement intake. China covers a vast territory and regions of different latitudes receiving different amounts of sunlight. Therefore, to evaluate the general vitamin D levels of the Chinese population, it is important to collect data from Eastern and Central China, such as Shandong and Henan Provinces, which are higher in elevation than Southern China, with thin air and less rainfall. In particular, Tengzhou City is located in the North China Plain in the north latitude of 34.5°, where the average annual number of hours of sunshine is 1,791, and Dengzhou City is located in the Nanyang Basin in the north latitude of 32.5°, where the average annual number of hours of sunshine reaches 1,935. Although another study has examined vitamin D levels by collecting data from several community centers (Lu, Zhang, et al., 2012), they used the immunoassay method to analyze vitamin D levels. Until now, immunoassays have been the most commonly used method to test 25(OH)D levels in China. However, the immunoassay methods are limited by the non‐equimolar detection of 25(OH)D_2_ and 25(OH)D_3_, antibody cross‐reactivity, and high inter‐laboratory variation (up to 38%) (Farrell et al., 2012; Lips, Chapuy, Dawson‐Hughes, Pols, & Holick, 1999; Singh, 2008). Considering these limitations of immunoassays, liquid chromatography‐tandem mass spectrometry (LC‐MS/MS) has become the gold standard for testing 25(OH)D levels (Mineva et al., 2015; Tai, Bedner, & Phinney, 2010). The LC‐MS/MS assay achieves higher sensitivity, specificity, and reproducibility compared to immunoassays and can determine 25(OH)D_2_ and 25(OH)D_3_ separately (Maunsell, Wright, & Rainbow, 2005). Vitamin D insufficiency is usually defined by 25(OH)D levels of ≤30 ng/ml, while a concentration of ≤20 ng/ml could represent vitamin D deficiency. Because of the various immunoassay methods, it is difficult to compare inter‐study data. There is currently no consensus on a cutoff value for confirming vitamin D deficiency (Holick et al., 2011; Ross et al., 2011; Yates, Schlicker, & Suitor, 1998).

Therefore, one of the goals of our study was to examine the prevalence of vitamin D insufficiency in pregnant women in Eastern and Central China and to evaluate all samples using the gold‐standard vitamin D LC‐MS/MS analysis method in a central laboratory. Additionally, we conducted a study to determine whether environmental factors would modify the link between 25(OH)D levels and SNPs during pregnancy to guide pregnant women in order to elevate their 25(OH)D levels.

## 2 METHODS

### 2.1 Study population

A total of 326 pregnant women who resided in Tengzhou and Dengzhou cities for at least 1 year were enrolled from several community centers between 2017 and 2019. Clinical information including age, weeks of gestation, body mass index (BMI), sun exposure, dietary and drinking habits, physical activities, and medical records during pregnancy was completed by all subjects.

### 2.2 Information and blood sample collection

The study protocol conformed to the ethical guidelines of the 1975 Declaration of Helsinki and was approved by the Medical Ethics Committee of Central People's Hospital of Tengzhou, Jining Medical University, and the Research Ethics Committee of the Central Hospital of Dengzhou. Informed consent was obtained from all participants. With the participants' consent, a fasting blood sample (8 hr) was drawn into a serum separator tube and an EDTA anticoagulant tube. Serum separator tubes were centrifuged (1,790 *g*, 5 min, 4°C) to separate serum (used for measuring 25(OH)D levels) and EDTA anticoagulant tubes used for DNA extraction. The participants' age, sex, height, and body weight were recorded. Other baseline characteristics such as the use of medication, smoking habits, level of physical activity, drinking habits, sleeping time, and lifestyle were obtained via a standardized questionnaire.

### 2.3 Single nucleotide polymorphisms

According to previous studies, relative SNPs have been selected in genes involved in vitamin D synthesis and metabolic pathways. In addition, these SNPs are located in the functional domain and play a vital role in vitamin metabolism from the NCBI database. The following were selected as candidate SNPs: VDR (rs10735810, rs3847987, rs1544410, rs7968585, rs2408876, rs10783219); GC (rs7041, rs4588, rs2282679, rs1155563, rs12512631, rs222020, rs16847015, rs17467825, rs2070741, rs2298849, rs16846876, rs842999, rs222035, rs3755967, rs11939173, rs2298850, rs12512631); CYP2R1 (rs2060793, rs10741657, rs1993116, rs7116978, rs12794714, rs1562902, rs10766197, rs10500804, rs10766197, rs10877012, rs11023374); CYP24A1 (rs6013897, rs927650, rs2248137, rs6068816, rs73913757, rs2209314, rs2762939, rs17470271); CYP27B1 (rs4646536, rs703842, rs10877012, rs118204009); CYP3A43 (rs680055, rs2242480); NADSYN1/DHCR7 (rs3829251, rs1790349, rs12785878, rs7944926, rs12800438, rs3794060, rs4945008, rs4944957); RXRA (rs9409929, rs11185644). A Rapid DNA Extraction and Detection Kit (Tiangen) was used to extract DNA from peripheral blood leukocytes according to the manufacturer's protocol, and SNP genotyping was conducted and analyzed using the MassARRAY iPLEX Gold platform (Sequenom).

### 2.4 Serum 25(OH)D laboratory measurements

The 25(OH)D_2_ and 25(OH)D_3_ levels in the serum were measured using a modified LC‐MS/MS method. Briefly, 100 μl aliquots of calibrators or serum samples were spiked with 500 μl isotope‐labeled internal standard solution. Samples were vortexed for 1 min and centrifuged for 5 min at 9,250 *g* and 4°C. After centrifugation, 400 μl of sample supernatant was transferred into a 96‐deep well plate and then dried under nitrogen at 60°C. Using 100 μl acetonitrile solution of PTAD, we performed derivatization for 60 min and then added 50 μl methanol for reconstitution. Subsequently, the residuals were injected into the UPLC‐MS/MS system (Waterscorporation). Chromatographic separation on the Waters ACQUITY UPLC I‐class was performed under binary gradient conditions with a mobile phase A of water with 0.1% formic acid (volume fraction) and 5 mM ammonium formate and mobile phase B of methanol with 0.1% formic acid. The column temperature was maintained at 40°C. Xevo TQD was used for MS/MS detection in positive electrospray ionization mode and multiple reaction monitor (MRM) mode. The accuracy of this method was validated by analyzing the National Institute of Standards and Technology SRM 972a. Comparing the measurement results with the reference values of SRM 972a, the accuracy of this method was 90%–115% and 95%–110% for 25(OH)D_2_ and 25(OH)D_3_, respectively.

### 2.5 Definitions

We used the commonly accepted cutoffs for severe 25(OH)D deficiency (<10 ng/ml), deficiency (10–20 ng/ml), and insufficiency (20–30 ng/ml). Levels ≥30 ng/ml were considered sufficient or optimum, while levels >150 ng/ml were considered vitamin D intoxication (Holick, 2007; Holick et al., 2011).

### 2.6 Statistical analysis

Statistical analysis was performed using the Statistical Package for Social Sciences (SPSS) 17.0 (SPSS Inc.) and R software. Student's *t* test or analysis of variance (ANOVA) was used to compare the mean values of continuous variables. Continuous variables were expressed as mean ± *SD*, while categorical variables were expressed as frequency and percentage. Body mass index was divided into four subgroups (BMI<18.5, 18.5≤BMI<24, 24≤BMI<28, and BMI≥28) according to the standard in China (Criteria of weight for adults, National Health and Family Planning Commission of the People's Republic of China, WS/T 428‐2011). Significant associations between selected SNPs and 25(OH)D levels were tested using the Kruskal–Wallis *H* test. The associations of SNPs with 25(OH)D deficiency were re‐analyzed using the Kruskal–Wallis *H* test modified by vitamin D supplementation and sunshine exposure. Differences were considered statistically significant at a *p* value <.05.

## 3 RESULTS

### 3.1 Characteristics of the study population

The study included 326 pregnant women based on biochemical tests, physical examinations, and detailed questionnaires. The levels of biochemical indicators and clinical characteristics are shown in Table 1. The average age was 29.65 ± 5.03 years, with a mean gestational age of 15.73 ± 2.16 weeks. The best part of the recruited women had a normal BMI (mean ± *SD*, 23.81 ± 3.85). Almost half of them experienced fewer physical activities (56.75%, ≤5 hr/week). Most of them had less sunshine exposure (98.77%, ≤4 hr/week) during pregnancy. None of the participants had drinking or smoking behavior during pregnancy.

**TABLE 1:** General characteristics of the participants

Variables	*n* (%)	Mean ± *SD*
Total of pregnant women	326 (100)	
25(OH)D (ng/ml)		
<10	95 (29.14)	14.57 ± 7.21
≥10– and <20	171 (52.45)	
≥20– and <30	45 (13.80)	
≥30	15 (4.60)	
Age, years		
0–25	62 (19.02)	29.65 ± 5.03
26–30	148 (45.40)	
31–35	77 (23.62)	
>35	39 (11.96)	
Gestational week, weeks		
≤12	66 (20.25)	15.73 ± 2.16
13–27	120 (36.81)	
≥28	18 (5.52)	
Pregnancy BMI, kg/m^2^		
BMI<18.5	10 (3.08)	23.81 ± 3.85
18.5≤BMI<24	174 (53.54)	
24≤BMI<28	137 (32.31)	
BMI≥28	36 (11.08)	
Physical activity (hr/week)		
≤5	185 (56.75)	4.17 ± 3.68
5–12	139 (42.64)	
>12	2 (0.61)	
Sun exposure (hr/week)		
≤4	322 (98.77)	0.98 ± 0.69
>4	4 (0.12)	
Smoker		
Yes	0 (0.00)	
No	326 (100.00)	
Drinker		
Yes	0 (0.00)	
No	326 (100.00)	
Vitamin D intake		
Yes	147 (45.09)	
No	179 (54.91)	

Abbreviations: 25(OH)D, 25‐hydroxyvitamin D; BMI, body mass index; *SD*, standard deviation.

### 3.2 Levels of 25(OH)D in pregnant women

Although nearly half of the participants had a habit of vitamin D intake (Table 1), we found that the average level of 25(OH)D among the participants was 14.57 ± 7.21 ng/ml (deficiency). Vitamin D severe deficiency and deficiency (<20 ng/ml) were found in 81.59% of pregnant women. Among them, 29.14% were seriously deficient. Very few pregnant women had sufficient levels of 25(OH)D (4.6%).

### 3.3 Correlation between 25(OH)D and other characteristics

To further determine the correlation between 25(OH)D levels and other related clinical characteristics, the influence factors including daily vitamin intake, age, body mass index (BMI), and physical activity were analyzed. As shown in Table 2, these data suggest that there was no significant correlation between 25(OH)D levels and age. The level of 25(OH)D was significantly associated with gestational week, BMI, physical activity, and vitamin D intake. Early pregnancy, lower BMI index, less physical activity, and vitamin D non‐intake were significantly associated with lower 25(OH)D levels.

**TABLE 2:** Serum 25(OH)D levels in participants according to their characteristics

Variables	25(OH)D levels (ng/ml) (mean ± *SD*)	*p* value
Age, years		
0–25	13.99 ± 6.02	.7898
25–35	14.72 ± 7.55	
>35	14.19 ± 6.18	
Gestational week, weeks		
≤12	12.42 ± 4.63	.0013**
13–27	16.55 ± 8.40	
≥28	16.37 ± 8.62	
Pregnancy BMI, kg/m^2^		
BMI<18.5	11.63 ± 4.37	.0068**
18.5≤BMI<24	13.55 ± 6.77	
24≤BMI<28	16.75 ± 7.92	
BMI≥28	15.22 ± 7.24	
Physical activity (hr/week)		
≤5	13.79 ± 6.85	.0347*
5–12	15.73 ± 7.48	
>12	19.11 ± 8.59	
Vitamin D intake		
Yes	18.04 ± 8.20	.0000***
No	11.72 ± 4.65	

* *p* < .05.

** *p* < .01.

*** *p* < .001.

### 3.4 Association between 25(OH)D levels and SNPs in the vitamin D pathway

To evaluate the possible contribution of variations in 25(OH)D status among pregnant women, 40 SNPs in the genes encoding the vitamin D pathway enzymes (VDR, GC, CYP2R1, CYP24A1, CYP27B1, CYP3A43, DHCR7, and RXR4) were analyzed. In the variance analysis, rs1155563 in GC and rs6013897 in CYP24A1 were associated with 25(OH)D levels among the pregnant women. Both SNPs with their minor alleles had lower 25(OH)D concentrations than those with major alleles (Table 3). No significant associations of other selected SNPs with vitamin D levels were observed.

**TABLE 3:** Association between single SNP and serum 25(OH)D level

Genotype	*N*	25(OH)D Mean ± *SD* (ng/ml)	*p*
rs1155563			
CC	44	11.73 ± 5.23	Ref
CT	62	13.58 ± 6.03	.104
TT	110	15.34 ± 7.68	.005**
rs6013897			
AA	63	9.60 ± 5.24	Ref
AT	44	13.98 ± 6.50	.023*
TT	168	14.94 ± 7.18	.008**

Note

Adjusted for age, pregnancy BMI, sun exposure, physical activity, and vitamin D supplements.

* *p* < .05.

** *p* < .01.

### 3.5 Effect of SNPs on 25(OH)D levels is influenced by vitamin D supplementation

We further classified Table 3 into two groups according to SNP genotype and type of the vitamin D supplement (Table 4). As shown in Table 4, the vitamin D supplement group (over 400 IU/day) had a significantly higher 25(OH)D level compared to the group without vitamin D supplements. Vitamin D supplementation eliminated the significant influence of genotypes (rs1155563 and rs6013897) on 25(OH)D levels in pregnant women. The pregnant women (without vitamin D supplement group) with rs1155563 and rs6013897 minor alleles had lower 25(OH)D levels, which were significantly associated with 25(OH)D levels.

**TABLE 4:** Association between SNPs and serum 25(OH)D concentration levels stratified by vitamin D supplements

Genotype	No vitamin D supplements			Vitamin D supplements		
	*n*	25(OH)D Mean ± *SD* (ng/ml)	*p*	*n*	25(OH)D Mean ± *SD* (ng/ml)	*p*
rs1155563						
CC	31	10.04 ± 3.45	Ref	23	15.78 ± 6.57	Ref
CT	41	12.16 ± 4.54	.028*	21	20.17 ± 7.78	.148
TT	47	12.71 ± 5.43	.015*	53	18.17 ± 8.73	.361
rs6013897						
AA	60	9.22 ± 2.52	Ref	33	10.83 ± 5.60	Ref
AT	65	11.91 ± 4.25	.052	43	18.33 ± 8.22	.121
TT	33	12.17 ± 5.27	.097	41	19.41 ± 7.03	.077

Note

Adjusted for age, pregnancy BMI, sun exposure, and physical activity.

* *p* < .05.

## 4 DISCUSSION

Low levels of vitamin D in pregnant women are associated with an increased risk of poor maternal and neonatal outcomes, including lower birth weight, gestational diabetes, and high blood pressure in the offspring (Chun, Shin, Kim, Joung, & Chung, 2017; Meems et al., 2016; Murthi et al., 2017). Although some studies have reported vitamin D deficiency in pregnant women in China (Foo et al., 2009; Hjelmesaeth et al., 2010; Lu, Zhang, et al., 2012), their results were limited due to the use of immunoassays.

One distinctive innovation of our research is the use of LC‐MS/MS to test the levels of 25(OH)D. We completed all test procedures in one single‐center analysis by a single experienced technician to eliminate the probability of inter‐laboratory variation. Based on this method, we identified that severe 25(OH)D deficiency or insufficiency was prevalent among pregnant women in Shandong and Henan Provinces, China. This finding is in line with previous studies, indicating that low 25(OH)D levels are a global problem (Arora, Goel, Chawla, Huria, & Arya, 2018; Holmes, Barnes, Alexander, McFaul, & Wallace, 2009). This study also found that the mean serum 25(OH)D level was 14.57 ± 7.21 ng/ml among the recruited pregnant women in Tengzhou, Dengzhou, Shandong, and Henan. More than 95.40% of pregnant women had 25(OH)D insufficiency and 81.59% of pregnant women had 25(OH)D deficiency. The dietary sources of vitamin D are mainly fat, fish, cod liver oil, and dairy products. However, the daily intake of fish and dairy products in Tengzhou and Dengzhou is usually insufficient. This study also revealed that most of them had less sunshine exposure (98.77%, ≤4 hr/week) during pregnancy. Insufficient vitamin D intake in the diet and sun exposure is major causes of vitamin D insufficiency in pregnant women in Tengzhou and Dengzhou. A previous study revealed that polymorphisms in GC, CYP3A4, and CYP24A1 genes were significantly associated with the bioavailability of 25(OH)D metabolism among pregnant women. CYP23A1 rs2209314 has also been associated with 25(OH)D levels (Duan, Xue, Ji, Zhang, & Wang, 2018; Shao et al., 2018). The findings of this study suggest that, although variants of rs1155563 in GC and rs6013897 in CYP24A1 were significantly associated with 25(OH)D levels among pregnant women in Eastern and Central China, vitamin D supplementation modified this effect. However, due to the limited number of samples, the variation of rs1155563 in GC and rs6013897 in CYP24A1 related to 25(OH)D levels requires further study. A more comprehensive study with a larger sample is required to validate our conclusions.

The main limitation of our study was its cross‐sectional design. Hence, the participants in our study were enrolled from Tengzhou and Dengzhou city, which do not encompass all people in Eastern and Central China. Therefore, selection bias was a problem in our study. Another limitation of the study was that the method we used (LC‐MS/MS) could not separately test all of the epimers of 25(OH)D, such as 3‐epi 25(OH)D_3_. However, due to their low concentration (Arora et al., 2018; Holmes et al., 2009), they are considered to have a negligible effect on total vitamin D levels.

In summary, the 25(OH)D status was tested using a reliable LC‐MS/MS method. The results indicate that pregnant women in Eastern and Central China are at high risk of vitamin D deficiency, suggesting the need for advanced training to improve food safety knowledge, attitudes, and practices. rs1155563 in GC and rs6013897 in CYP24A1 influenced 25(OH)D levels in pregnant women. This study also revealed that 25(OH)D levels were influenced by BMI, physical activity time, and vitamin D intake among pregnant women. Repletion in this population may require vitamin D dietary supplementation, increasing physical activity, and appropriate BMI. To improve the vitamin D nutritional status of pregnant women, the dosage and dietary structure of pregnant women's vitamin D should be appropriately determined.

## CONFLICT OF INTEREST

The authors declare that they do not have any conflict of interests.
